# Therapeutic drug monitoring of oral targeted antineoplastic drugs

**DOI:** 10.1007/s00228-020-03014-8

**Published:** 2020-11-09

**Authors:** Anna Mueller-Schoell, Stefanie L. Groenland, Oliver Scherf-Clavel, Madelé van Dyk, Wilhelm Huisinga, Robin Michelet, Ulrich Jaehde, Neeltje Steeghs, Alwin D.R. Huitema, Charlotte Kloft

**Affiliations:** 1 Dept. of Clinical Pharmacy and Biochemistry, Institute of Pharmacy, Freie Universitaet Berlin, Berlin, Germany; 2 Graduate Research Training Program, PharMetrX, Berlin/Potsdam, Germany; 3 Department of Clinical Pharmacology, Division of Medical Oncology, The Netherlands Cancer Institute—Antoni van Leeuwenhoek, Plesmanlaan 121, 1066 CX Amsterdam, The Netherlands; 4 Institute of Pharmacy and Food Chemistry, Julius-Maximilians-Universität Würzburg, Würzburg, Germany; 5 College of Medicine and Public Health, Flinders University, Adelaide, SA Australia; 6 Institute of Mathematics, University of Potsdam, Potsdam, Germany; 7 Department of Clinical Pharmacy, Institute of Pharmacy, University of Bonn, Bonn, Germany; 8 Department of Pharmacy & Pharmacology, The Netherlands Cancer Institute—Antoni van Leeuwenhoek, Amsterdam, The Netherlands; 9 Department of Clinical Pharmacy, University Medical Center, Utrecht University, Utrecht, The Netherlands

**Keywords:** Targeted antineoplastic drugs, Tyrosine kinase inhibitors, Therapeutic drug monitoring, Oral anticancer drugs, Personalised medicine

## Abstract

**Purpose:**

This review provides an overview of the current challenges in oral targeted antineoplastic drug (OAD) dosing and outlines the unexploited value of therapeutic drug monitoring (TDM). Factors influencing the pharmacokinetic exposure in OAD therapy are depicted together with an overview of different TDM approaches. Finally, current evidence for TDM for all approved OADs is reviewed.

**Methods:**

A comprehensive literature search (covering literature published until April 2020), including primary and secondary scientific literature on pharmacokinetics and dose individualisation strategies for OADs, together with US FDA Clinical Pharmacology and Biopharmaceutics Reviews and the Committee for Medicinal Products for Human Use European Public Assessment Reports was conducted.

**Results:**

OADs are highly potent drugs, which have substantially changed treatment options for cancer patients. Nevertheless, high pharmacokinetic variability and low treatment adherence are risk factors for treatment failure. TDM is a powerful tool to individualise drug dosing, ensure drug concentrations within the therapeutic window and increase treatment success rates. After reviewing the literature for 71 approved OADs, we show that exposure-response and/or exposure-toxicity relationships have been established for the majority. Moreover, TDM has been proven to be feasible for individualised dosing of abiraterone, everolimus, imatinib, pazopanib, sunitinib and tamoxifen in prospective studies. There is a lack of experience in how to best implement TDM as part of clinical routine in OAD cancer therapy.

**Conclusion:**

Sub-therapeutic concentrations and severe adverse events are current challenges in OAD treatment, which can both be addressed by the application of TDM-guided dosing, ensuring concentrations within the therapeutic window.

**Supplementary Information:**

The online version of this article (10.1007/s00228-020-03014-8) contains supplementary material, which is available to authorized users.

## Introduction

With the approval of imatinib in 2001 [1], kinase inhibitors (KIs) have significantly improved the prognosis of many cancers. As of April 2020, 71 oral antineoplastic drugs (OADs) targeting a large assortment of molecular targets (Supplementary Fig. 1) are approved by the European Medicines Agency (EMA) and/or the US Food and Drug Administration (FDA).

With more OADs available, both the route of administration and the treatment setting are changing. While i.v. chemotherapy is mainly administered in an in-patient setting, OADs allow outpatient care with both its advantages and disadvantages. Strong advantages are the level of independence, and, due to outpatient treatment, a reduction of health care costs. At the same time, the responsibility for adhering to treatment schedules is moved to the patient. Given the often-complex treatment regimens, patients must be well trained and motivated to take their medication correctly. Moreover, patients should have knowledge on the frequency and severity of possible adverse events (AEs) and on preventive and responsive measures to limit them. Yet, adherence to targeted OADs is variable.

Other aspects to consider are the complex pharmacokinetics (PK) of OADs [2]. Although the right drug (‘what’) is increasingly selected based on the tumour characteristics, a fixed dose (‘how much’) is mostly given in OADs, leading to large differences between individual plasma concentrations. High interindividual variability (IIV) in exposure at standard dosing, mostly ranging from 19 to 100% [3] and up to 16-fold for gefitinib [4], has been described for OADs. While modern phase I studies increasingly assess exposure-response relationships and maximum tolerated doses (MTD) become harder to identify, the fixed dose for a new antineoplastic drug is historically established in a phase I study using a 3 + 3 design, which focuses on toxicity [5]. The MTD, defined as the dose level below the toxic dose level, is usually adopted as the recommended phase II dose [5]. Few patients participate in phase I trials (median *n* = 26 [6]) which limits the generalisability of the selected dose. Based on the lack of focus on efficacy, a proportion of patients will show sub-therapeutic plasma concentrations [7] and be at risk for treatment failure at the early determined MTD. At the same time, some patients will show toxic plasma concentrations and thus an increased risk for non-adherence [8] as consequence of AEs [9].

One strategy to prevent sub-optimal drug concentrations is the use of therapeutic drug monitoring (TDM), i.e. dosing based on measured drug exposure [10], guiding OAD dosing [7, 11–14]. By tailoring drug doses to individual patients, the proportion of patients with sub-optimal drug concentrations can be reduced. TDM has already been well-adopted in other therapeutic areas such as antimicrobial and antiepileptic therapy [15–17]. Despite its value in oncology becoming more recognised [18–21], it is still not commonly used in antineoplastic treatment.

In the following sections, we elaborate on the unexploited value of TDM in OAD therapy. After introducing various forms of TDM and TDM for OADs specifically, an overview of current evidence for drug target concentrations is provided. Moreover, we describe available PK models, observed PK exposure, TDM targets and data on exposure-response and exposure-safety relationships for OADs that are approved by at least one regulatory agency. Finally, TDM recommendations are given for OADs, for which targets were established and TDM has proven feasible.

## Therapeutic drug monitoring

Therapeutic drug monitoring (TDM) refers to measuring drug concentrations to assess if drug concentrations are within the therapeutic target range and, if necessary, individualise dosing regimens. An unpredictable dose-exposure relationship, a small therapeutic window with a defined target concentration, a high PK and/or pharmacodynamic (PD) IIV and nonlinear PK are best indicators for a benefit from TDM [7, 22]. The absence of an exposure-response relationship and high intraindividual and interoccasion (IOV) PK/PD variability relative to the IIV are characteristics of drugs unsuitable for TDM [23, 24] (Supplementary Fig. 2). Several forms of dose individualisation exist. These are classified as a priori and a posteriori approaches, depending on the level of individualisation before treatment initiation [15]. In an a priori framework, information on both drug and patient characteristics are used to guide initial dosing [25]*.* Based on established relationships between patient characteristics and PK parameters, initial dosing can be individualised to patient sub-populations [26]. However, no individual PK information is included in an a priori framework, resulting in moderate average bias and precision [26].

Individual drug concentrations obtained after treatment start are used in a posteriori TDM [15]. Following the detection of non-optimal drug concentrations, different procedures for dose adjustments are possible: in the simplest case, oncologists will use the drug label, dosing algorithms or nomograms to determine a new dose [27]. Although simple, this approach requires to abide with the scheduled blood sampling times and is unsuitable if the patient is not represented by the population on which drug label or dosing algorithm have been developed on [28].

Another a posteriori approach involves the collection of 4–8 blood samples within a dosing interval and the subsequent calculation of the area under the concentration-time curve (AUC) [11]. Based on the calculated AUC, individual PK parameters can be obtained and used for PK calculations to determine a more suitable dose. However, dense blood sampling is rarely feasible in clinical practice [29].

Population PK (nonlinear mixed-effects) modelling and simulation [30] can aid in optimising TDM in multiple ways: first, PK information from the population can be incorporated into model parameters during model development. Use of this information allows to refrain from dense blood sampling in model-informed precision dosing (MIPD) and often few samples are enough to obtain sufficiently precise individual PK estimates [26]. Second, sampling at fixed time points is no longer necessary and can also be performed prior to steady-state attainment [26]. As long as actual sampling times are documented, samples from virtually every time point can be used for PK analyses in MIPD [26]. Still, there are more and less informative sampling time points. Optimal design, another part of the model-informed dose individualisation process, can aid in systematically determining the most informative sampling time point(s) within a given time frame [31]. Finally, Bayesian TDM in MIPD combines model-informed TDM with the ability to learn and subsequently forecast drug concentrations at various possible dosing regimens. Similarly to traditional population PK, the Bayesian approach uses information from the population to estimate the most likely PK parameter values for a given drug and population [32]. If specific patient characteristics influence one or more of the PK processes, this information can already be used in an a priori dose selection process. At the beginning of treatment, when no concentration measurements are available, predicted PK parameter values for a specific patient will be identical with the population estimates [29]. As measured drug concentrations become available, they are used to refine the patient’s predicted PK parameter values. The more patient-individual information (i.e. drug concentrations) is available, the more weight is set on this information in the parameter estimation process and the more individual parameter estimates will be allowed to deviate from the population estimates [29]. Moreover, Bayesian TDM can account for IOV that is lower than the safe and effective variability [33] and still predict future doses based on at least two sampling occasions [34, 35]. A disadvantage of Bayesian TDM is the high shrinkage of predicted individual PK parameters if only a single PK sample is available: when the population outweighs the individual information, individual information on the patient will get lost as the empirical Bayes estimates shrinks to the typical population parameters [29]. Moreover, applying Bayesian TDM requires special knowledge, can be time intensive and thus difficult to implement in clinical practice.

Sampling minimum plasma concentrations at steady-state (C_min,ss_) is often performed in clinical practice and, if done correctly, the currently most precise approach as it avoids shrinkage of individual information to the population mean. However, it requires precise information about the patient’s dosing schedule and good coordination between patient and treatment team. An easy and time-efficient way to circumvent the need to sample at C_min,SS_ is to account for the difference between the time of minimum concentrations and time of measurement and extrapolate based on the time after last dose and the terminal half-life of the drug. In this method, based on an algorithm described and validated for imatinib [10], samples can be taken at random time points in the elimination phase of the drug and the corresponding C_min,SS_ can be calculated using Eq. (1).

1 $$ {C}_{\min, \mathrm{SS}}={C}_{\mathrm{measured}}\ast {0.5}^{\frac{\mathrm{Dosing}\ \mathrm{Interval}\ \left[\mathrm{h}\right]-\mathrm{Time}\ \mathrm{after}\ \mathrm{last}\ \mathrm{dose}\left[\mathrm{h}\right]}{\mathrm{Half}-\mathrm{life}\ \left[\mathrm{h}\right]}} $$

Of note, this method assumes that C_measured_ is sampled in the terminal phase of a monoexponential decline. For drugs with a nonlinear clearance or a short half-life (i.e. dasatinib, axitinib), an alternative method has to be used. For example, the C_min,SS_ can also be estimated based on a randomly taken concentration measurement (C_measured_) and a simulated typical concentration-time curve, using an existing population PK model. Based on the ratio of the measured concentration at t_measured_ with the concentration in the simulated PK profile, the corresponding C_min,SS_ in this patient can be estimated [36].

## Therapeutic drug monitoring for oral targeted antineoplastic drugs

Several OAD characteristics suggest individualised dosing:

1. OADs show highly variable drug exposure, caused by IIV in absorption, distribution, metabolism and excretion (ADME). Oral bioavailability (BA) differs between and within agents (i.e. 14–34% in dasatinib and 98% in imatinib) and depends on drug formulation [37], absorption, first-pass hepatic metabolism and food intake. Moreover, almost all OADs are metabolised by monooxygenases of the Cytochrome P450 (CYP) family [3]. Up to 20-fold variability in expression and activity of CYP3A4 has been reported, and polymorphisms in the isoenzymes CYP2D6, CYP2C9 and CYP2C19 additionally contribute to the variable metabolic activity [3]. The activity of CYP enzymes may be additionally influenced by concomitant administration of CYP inducers/inhibitors, environmental factors, smoking and food intake [3, 38]. Polymorphic transporters are also involved in the excretion of many agents (i.e. axitinib, dasatinib and sorafenib) [3].
2. Efficacy is challenging to assess during OAD treatment, as benefits in clinical outcome parameters such as overall survival (OS) and progression-free survival (PFS) take long until evaluable. Objective response rates using CT scans can be assessed earlier and for a few malignancies, reliable biomarkers are available (i.e. prostate specific antigen for prostate cancer or complete cytogenic response (CCyR) for chronic myeloid leukaemia (CML)). Furthermore, advances in PKPD modelling allow to use tumour dynamics in exposure-response analyses [39]. If an exposure-response relationship has been established, achieving target concentrations can serve as a proxy for achieving beneficial outcomes.

As disease progression can be fatal in oncology, treatment at an exposure above the efficacy threshold should be assured from the start of treatment or at least achieved as soon as possible, while individual patient toxicity should be monitored carefully. Furthermore, dose increases should only be implemented in case of acceptable toxicity and patients with low exposure and considerable toxicity should be switched to another treatment option.

Of note, while TDM might be crucial for agents with a narrow therapeutic window (i.e. pazopanib, sunitinib), it might be less relevant for agents with a wider therapeutic window (i.e. erlotinib, osimertinib).

Considering the high costs of OADs, cost-neutral PK-guided dose interventions to increase exposure, i.e. concomitant intake with food [40–42], split intake moments [43] or boosting (i.e. with a CYP3A4 inhibitor), should be considered before conventional dose increments, particularly in countries with poor healthcare systems.

For some agents, TDM has already proven feasible [44]. Strong evidence exists for imatinib in CML [18, 45] and gastrointestinal stromal tumours (GIST) [46]. Additional compounds for which TDM was feasible in prospective studies are sunitinib [47], pazopanib [48], tamoxifen [20] and abiraterone [40]. For other agents, i.e. alectinib [49], axitinib, crizotinib [49], trametinib [50] and vemurafenib [51–54], a PK target associated with either efficacy or toxicity has been established, but not yet evaluated in prospective clinical studies [13]. Lastly, no information about the value of TDM is available for some compounds. Most of these are new, and exposure-response relationships have not been established yet. For those drugs, we suggest to target the mean/median exposure as proxy for a PK target, as previously established PK targets amounted to 85% (± 19%) [14] and 82% (± 17%) [13] of the mean population exposures in AHDs and KIs, respectively. This is already applied in the DPOG-TDM study [55] and similar approaches are suggested by the FDA for special populations [56–58]. In the DPOG-TDM study [55], the feasibility, tolerability and efficacy of TDM for 23 different OADs is currently being evaluated (www.trialregister.nl; NL6695)) and preliminary results are promising [59].

The rapid improvement in OAD treatment together with the continuous development of new compounds poses a challenge for the timely establishment of viable TDM targets. While exposure-safety relationships are determined early during drug development, observing exposure-response relationships requires extensive time. Accordingly, there is often a discrepancy between the level of viability of proposed PK/PD targets and the clinical relevance of a compound. For example, while imatinib was approved in 2001 [1], the exposure-response relationships in CML and GIST became publicly available in 2008 [45] and 2009 [46], respectively. Sunitinib was first approved in 2006, but the exposure-response relationship was published in 2010 [60]. Likewise, pazopanib was approved by the EMA in 2010 and the exposure-response relationship was first described in 2014 [61].

In the following section, we explore the potential of TDM-guided dosing to optimise OAD treatment. For each drug, we searched PubMed and Google Scholar using the terms ‘pharmacokinetics’, ‘exposure response’, ‘exposure efficacy’, ‘exposure safety’, ‘exposure toxicity’, ‘therapeutic drug monitoring’ and ‘TDM’ together with the respective drug name. Additionally, we reviewed the respective EMA European Public Assessment Reports and the FDA Clinical Pharmacology and Biopharmaceutics Reviews. In Supplementary Tables 1a-c, KIs, AHDs and other OADs are summarised together with their molecular target(s), therapeutic indication and date of first approval. Table 1 presents current evidence for TDM-guided dosing of OADs. In this table, each drug is classified according to the level of evidence currently available for TDM. If there is an established exposure-response relationship and a PK target, TDM is considered potentially useful. If additionally, a feasibility study has been performed, TDM is recommended. If on top of that, randomised, prospective studies demonstrated a positive effect of TDM, it is strongly recommended. If there is no evidence for an exposure-response relationship, TDM is considered exploratory. If there are minimal data on the PK of a drug, there are more useful targets than plasma concentration or there is evidence that TDM is not useful, it is not recommended. Compounds with the highest clinical relevance as monotherapy and for which TDM is classified as recommended are discussed below.

**Table 1:**
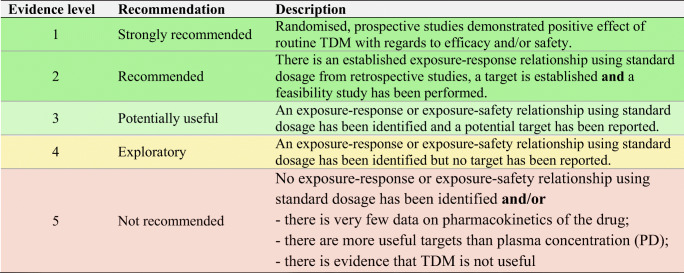

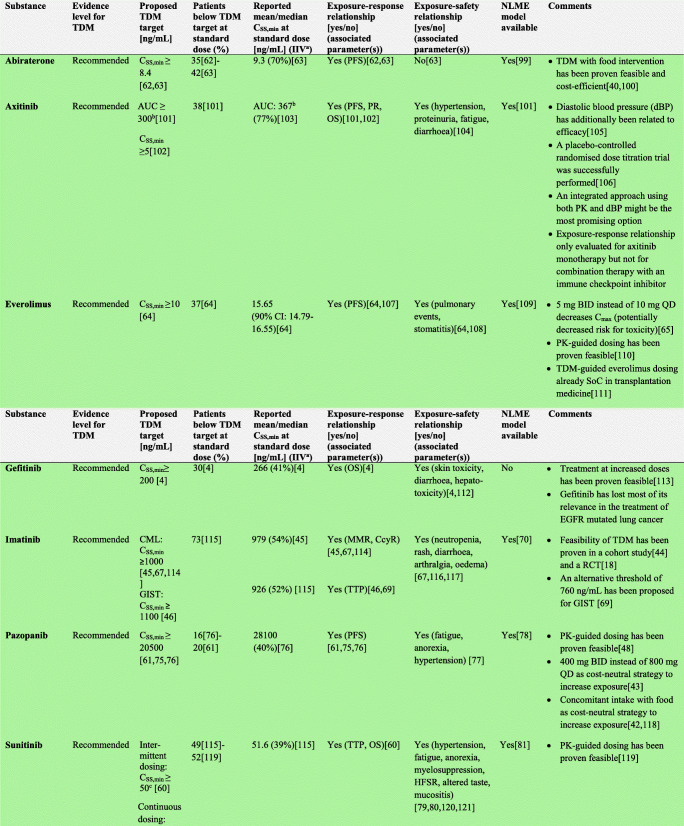

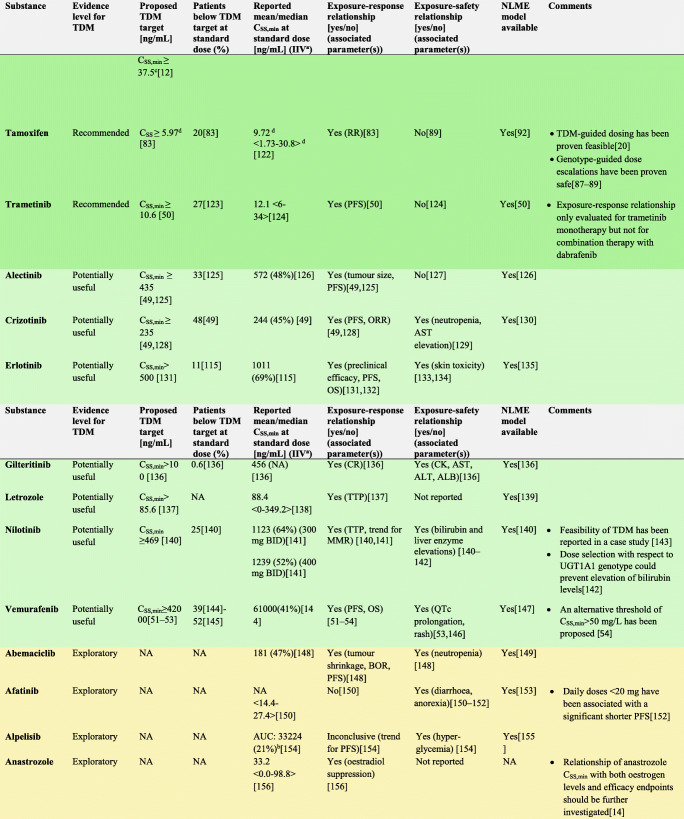

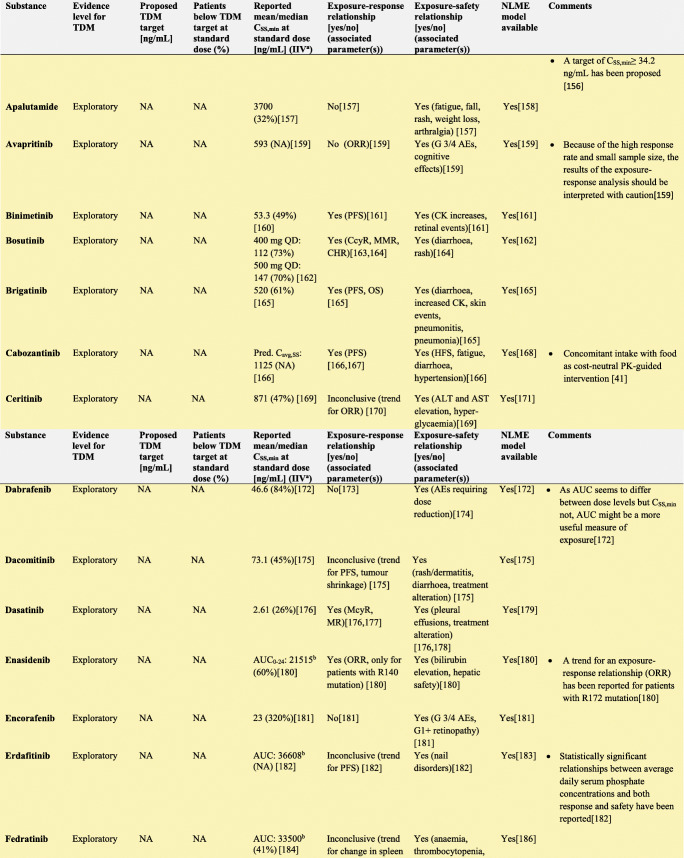

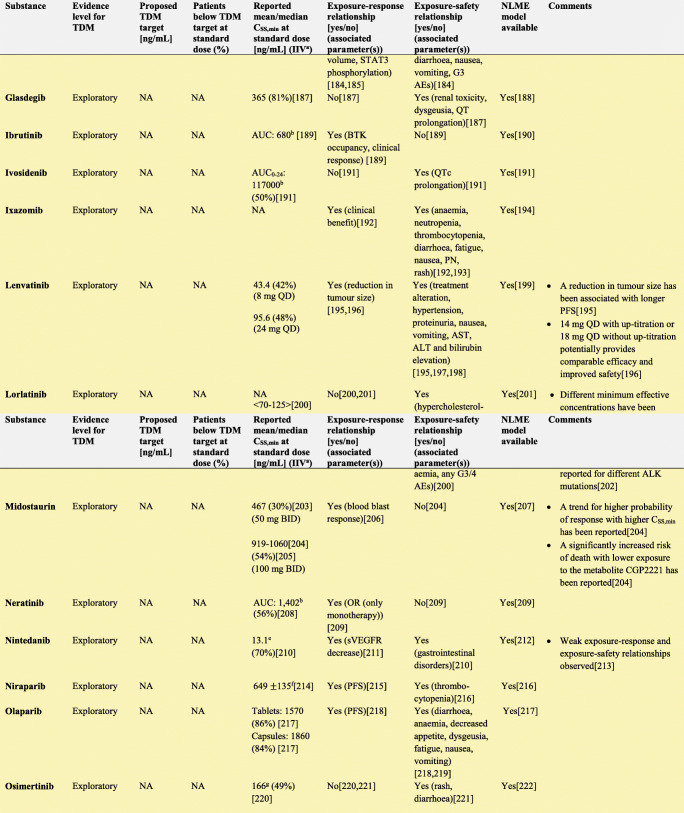

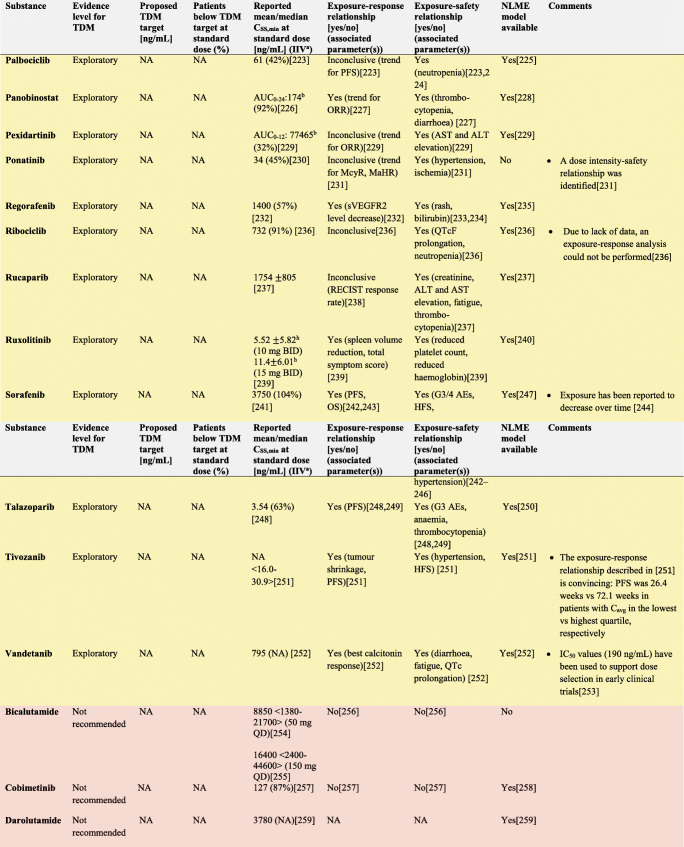

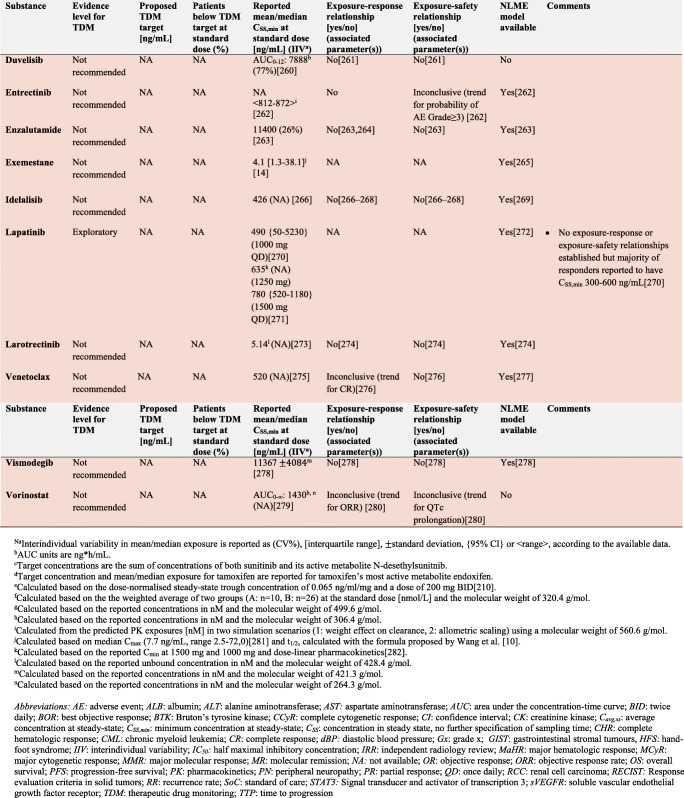
Evidence for TDM for targeted oral antineoplastic drugs

### Abiraterone

In an observational study in 61 metastatic castration-resistant prostate cancer patients, C_min_ ≥ 8.4 ng/mL were associated with a significantly longer PFS compared to C_min_ < 8.4 ng/mL (PFS 7.4 vs 12.2 months, *p* = 0.044) [62]. This threshold was later confirmed in a real-world patient cohort (*n* = 62, PFS 6.1 vs. 16.9 months, *p* = 0.033) [63]. Yet, at the standard dose of 1000 mg once daily (QD), 35% and 42% of patients, respectively, did not reach this target [62, 63]. A prospective study (*n* = 32) demonstrated that 20 patients (63%) had at least one C_min_ < 8.4 ng/mL with standard care [40]; however, when a light meal or snack was concomitantly taken with abiraterone, adequate exposure in 28 patients (87.5%) without additional toxicities was achieved [40]. Thus, TDM of abiraterone and concomitant food intake as a cost-neutral PK-guided intervention to reach C_min_ > 8.4 ng/mL has proven feasible. Given the absence of an exposure-toxicity relationship, a pragmatic option could be to include concomitant food intake in the drug label.

### Everolimus

In a meta-analysis, it has been reported that a two-fold increase in C_min_ was linked to an increased reduction in tumour size and C_SS,min_ ≥ 10 ng/mL could be used as a cut-off value [64]. At the same time, C_SS,min_ > 26.3 ng/mL have been associated with a 4-fold increased risk of toxicity compared to C_SS,min_ < 26.3 ng/mL [21]. As the occurrence of AEs seemed to be associated with high maximum concentrations (C_max_) [65], Verheijen et al. investigated the potential of alternative dosing to reduce C_max_-related AEs while maintaining therapeutic C_SS,min_._._ In a crossover study in 11 patients, administering 5 mg twice daily (BID) instead of 10 mg QD significantly reduced everolimus C_max_ while C_SS,min_ increased from 9.6 to 13.7 ng/mL [65]. Given the established exposure-response and exposure-toxicity relationships, we propose to combine 5 mg BID dosing with TDM to target a therapeutic window of C_SS,min_ ≥ 10 ng/mL and < 26.3 ng/mL. The developed population PK model by Combes et al. [66] could serve as a starting point in a MIPD framework.

### Imatinib

Higher frequencies of CCyR and major molecular response (MMR) have been reported in CML patients with high imatinib C_min,SS_ [45, 67]. Current evidence supports the use of a C_min,SS_ ≥ 1000 ng/mL as PK target to achieve improved CCyR and MMR in CML [68]. Imatinib C_min,SS_ > 3000 ng/mL have been associated with higher rates of AEs [67]. Therefore, a therapeutic window of 1000 ≤ C_min,SS_ < 3000 ng/mL seems reasonable [68]. In gastrointestinal stromal tumours (GIST), one study determined a longer time to disease progression in patients (*n* = 73) with C_SS,min_ ≥ 1100 ng/mL [46]. In another study, a significantly longer PFS was found in patients with C_min,SS_ ≥ 760 ng/mL compared to patients with C_min,SS_ < 760 ng/mL (PFS not reached vs. 56 months, respectively), although this patient population was not representative of routine clinical practice [69]. The feasibility of TDM-guided dosing to achieve imatinib C_min,SS_ of 750–1500 ng/mL has been proven in a prospective randomised controlled trial [18], and several population PK models [70–72] are available for use in MIPD of imatinib. As the fraction of patients reaching durable C_min,SS_ ≥ 1000 ng/mL has been reported to be as low as 33.3% [73], individualised imatinib dosing is highly relevant. As imatinib C_min_ have been reported to decrease during the first 3 months of treatment [74], it is important to keep measuring imatinib C_min,SS_ during treatment and after dose adjustments.

### Pazopanib

An association of C_SS,min_ ≥ 20.5 mg/L with improved PFS (19.6 vs. 52.0 weeks, *p* = 0.004) and tumour shrinkage was found in a retrospective analysis in 177 patients with advanced renal cell carcinoma (RCC) [61]. This efficacy threshold was later validated in the adjuvant setting [75] and in a real-life patient cohort [76]. However, 16–20% [61, 76] of patients do not reach this threshold and are thus at risk of decreased efficacy. In a prospective feasibility study of individualised pazopanib dosing, 57% of all patients (*n* = 30) showed pazopanib C_SS,min_ < 20 mg/L under standard treatment and 41% of these successfully achieved therapeutic C_SS,min_ upon dose increases to 1000–1800 mg QD [48]. Furthermore, all patients who achieved a partial response showed C_SS,min_ ≥ 20 mg/mL. In a recent retrospective observational clinical study in 27 RCC patients, a significant correlation between pazopanib C_SS,min_ ≥ 20.5 mg/mL and objective response was established [77]. Based on the evidence for an exposure-response relationship and the proven feasibility of individualised dosing, we recommend TDM-guided pazopanib dosing, targeting plasma C_SS,min_ ≥ 20 mg/mL. A published population PK model [78] can be used in a MIPD framework for pazopanib. However, due to a dose-dependent decrease in the relative BA of pazopanib, conventional dose increases are an inefficient strategy to increase exposure. Alternative cost-neutral strategies have been described in literature. Splitting intake moments (i.e. 400 mg BID instead of 800 mg QD) resulted in a 79% increase in C_min_ [43]. Moreover, concomitant intake with food successfully increased exposure as well [42].

### Sunitinib

Significant increases in toxicities in patients with sunitinib + active metabolite SU012662 C_SS,min_ ≥ 100 ng/mL have been reported [79, 80]. For RCC patients, an efficacy PK target of 50–100 ng/mL has been proposed in intermittent dosing at 50 mg QD [80]. Exploiting dose linearity, this target was extrapolated to C_SS,min_ ≥ 37.5 ng/mL for continuous dosing at 37.5 mg QD in GIST patients [12]. Based on a summary of exposure-response analyses [60], TDM-guided sunitinib dosing targeting a sunitinib + SU012662 C_SS,min_ of 50–100 ng/mL was prospectively tested in a clinical study in 43 patients with advanced solid malignancies [47]. Of the patients eligible for PK-evaluation (*n* = 29), 52% (*n* = 14) showed sunitinib + SU012662 C_SS,min_ < 50 ng/mL at treatment initiation, and among those, 5 patients reached therapeutic total trough levels after dose escalation without experiencing additional toxicities. These findings underline both the need and feasibility of TDM-guided sunitinib dosing, for which a published population PK/PD model can be used [81]. Biomarkers such as the soluble vascular endothelial growth factor receptor may provide additional information on individual response and have been integrated into PK/PD models [82]. Because of the different half-lives of sunitinib and SU012662, C_SS,min_ should be calculated separately when using the log-linear extrapolation method. Due to the long half-lives and time to reach steady-state, it is important to collect PK samples in the last treatment week before the off-treatment period.

### Tamoxifen

Compared to higher values, C_SS_ of < 5.97 ng/mL [83] and < 5.2 ng/mL [84] of tamoxifen’s active metabolite endoxifen have been associated with more additional breast cancer events and shorter distant relapse-free survival, respectively. While body weight and age have a significant impact as well [85], CYP2D6 phenotype accounts for 18–43% of the observed IIV of 40–49% in endoxifen C_SS_ [14]. Considering this, TDM of endoxifen might be promising to identify patients with sub-optimal target concentrations [86]. Because no toxic tamoxifen dose has been identified, dose increases up until 120 mg QD for patients with endoxifen C_SS_ < 5.97 ng/mL have been investigated and TDM has proven feasible [20, 87–92]. As it takes about 3 months to attain endoxifen steady state, we propose to use MIPD for early endoxifen target attainment [92].

## Discussion

While exposure-response and exposure-safety relationships have been observed for many OADs, viable PK targets are only available for a few. Future clinical and ‘real-world’ studies are needed to identify clear target ranges associated with favourable outcome. More PK/PD analyses conducted in (pre-)clinical development could help to characterise exposure-response relationships earlier. More focus must also be dedicated on the establishment of TDM as part of routine patient care. This might be challenging, as bio-analytical assays should be available and a solid logistic system with a short turn-around time in place. At the Netherlands Cancer Institute, TDM has been implemented in routine care, and PK samples are collected at routine visits to the outpatient clinic. Concentrations of 35 different OADs are measured weekly using liquid chromatography-tandem mass spectrometry [93–96], treatment recommendations are reported within 24 h to the treating physician and results can be discussed with patients 1–2 weeks after their visit. This approach is also emerging in Australia with several OADs being measured on request with current efficacy-implementation studies underway. Additional data should be prospectively collected (i.e. in registries) to further investigate the effect of TDM on treatment outcomes. Novel microsampling techniques, i.e. volumetric absorptive microsampling (VAMS) [97], could help to provide the TDM results even before patients visit the outpatient clinic. VAMS allows to precisely sample a small volume of capillary blood from the fingertip with a dedicated sampling device. After blood collection, the device is dried in the open air and shipped to a laboratory via regular mail without pre-processing or cooling during transport. Given its easy and minimally invasive character, this technique shows high potential: in the future, VAMS samples could be obtained at home and shipped to a laboratory by patients themselves. Upon sample analysis, results would be communicated to the treating oncologist and discussed with the patient at the next visit. Of note, the disadvantages of VAMS are not fully elucidated yet. Current limitations are variable analyte recoveries dependent on haematocrit [98] and the time-consuming determination of capillary-to-venous blood conversion factors, needed to compare measured capillary whole blood with venous plasma target concentrations [97]. Furthermore, to make this approach feasible, a well-connected infrastructure of oncologists, laboratories and PK-specialists must be available.

## Conclusion

In this review, we summarised the opportunities and challenges associated with TDM of OADs and outlined different TDM approaches, their respective advantages and disadvantages. We provided strong arguments why routine TDM should be established as a part of OAD treatment and reviewed the available evidence for all oral targeted antineoplastic drugs currently approved by the EMA and/or FDA. Finally, we provided an outlook into the future and proposed a strategy to increase feasibility and acceptance of TDM as part of routine clinical care.

## Supplementary Information

ESM 1 (DOCX 746 kb)

## Data Availability

Not applicable.
