# Supplementary material for: Therapeutic drug monitoring of oral targeted antineoplastic drugs
Source: Eur J Clin Pharmacol. 2020 Nov 9;77(4):441–64. doi: 10.1007/s00228-020-03014-8 (PMC7935845; doi:10.1007/s00228-020-03014-8)
Supplement: Supplementary file 1 — (DOCX 746 kb) [file 228_2020_3014_MOESM1_ESM.docx]

**Electronic supplementary material**

**Therapeutic drug monitoring of oral targeted antineoplastic drugs**

Authors: Anna Mueller-Schoell, Stefanie L. Groenland, Oliver Scherf-Clavel, Madlé van Dyk, Wilhelm Huisinga, Robin Michelet, Ulrich Jaehde, Neeltje Steeghs, Alwin D.R. Huitema, Charlotte Kloft

Corresponding Author: Charlotte Kloft, [charlotte.kloft@fu-berlin.de](mailto:charlotte.kloft@fu-berlin.de)

**Supplementary Table 1a: Overview of protein kinase inhibitors (KIs)**

| Compound name | Compound class | Molecular targets | Indication | Approved dose | First approval [year] |
| --- | --- | --- | --- | --- | --- |
| Abemaciclib | CDK4/6 inhibitor | CDK4/6 | BC | 150 mg BID | 2018 (EMA, FDA) |
| Afatinib | EGFR inhibitor | EGFR, HER2, HER4 | NSCLC (EGFR mut.) | 40 mg QD | 2013 (EMA, FDA) |
| Alectinib | ALK inhibitor | ALK, RET | NSCLC (ALK rear.) | 600 mg BID | 2015 (FDA), 2017 (EMA) |
| Alpelisib | PI3K inhibitor | PIK3α | BC | 300 mg QD | 2019 (FDA), Pending (EMA) |
| Avapritinib | KIT & PDGFRα inhibitor | PDGFRα, KIT | GIST | 300 mg QD | 2020 (FDA; OD), Pending (EMA) |
| Axitinib | VEGF inhibitor | KIT, PDGFRβ, VEGFR1-3 | RCC | 5 mg BID | 2012 (EMA, FDA) |
| Binimetinib⸹ | MEK inhibitor | MEK1-2 | MEL (BRAFV600+) | 45 mg BID | 2018 (EMA, FDA) |
| Bosutinib | BCR-ABL inhibitor | BCL-ABL, Src | CML (Ph+) | Newly diagnosed CP: 400 mg QD  CP-, AP- or BC with resistance to prior therapy: 500 mg QD | 2012 (FDA), 2013 (EMA) |
| Brigatinib | ALK inhibitor | ALK, ROS1, IGF-1R | NSCLC (ALK rearr.) | 90 mg QD (first 7 days)  180 mg QD (after day 7) | 2018 (EMA) |
| Cabozantinib | VEGF inhibitor | VEGFR2, FLT3, KIT, c-MET, RET | RCC, HCC  MTC | 60 mg QD (tablets, RCC) 140 mg QD (capsules, HCC/MTC) | 2012 (FDA; OD), 2014 (EMA; OD) |
| Ceritinib | ALK inhibitor | ALK, IGF-1R, ROS1 | NSCLC (ALK rearr.) | 450 mg QD | 2014 (FDA), 2015 (EMA) |
| Cobimetinib⸸ | MEK inhibitor | MEK1-2 | MEL (BRAFV600+) | 60 mg QD for 3 weeks on, 1 week off | 2015 (EMA, FDA) |
| Crizotinib | ALK inhibitor | ALK, c-MET, ROS1 | NSCLC (ALK rearr.), NSCLC (ROS1+) | 250 mg BID | 2011 (FDA), 2012 (EMA) |
| Dabrafenib† | BRAF inhibitor | B-Raf | ATC, MEL, NSCLC  (all BRAFV600+) | 150 mg BID | 2013 (EMA, FDA) |
| Dacomitinib | EGFR inhibitor | EGFR | NSCLC (EGFR mut.) | 45 mg QD | 2018 (FDA), 2019 (EMA) |
| Dasatinib | BCR-ABL inhibitor | BCR-ABL kinase, Src, KIT, PDGFRα/β, EphA2 | ALL (Ph+)  CML (Ph+) | 140 mg QD  CP: 100 mg QD  AP, MP, BP: 140 mg QD  Children (CML & ALL):  40 mg (10-19 kg) 60 mg (20-29 kg) 70 mg (30-44 kg) 100 mg (≥45 kg) | 2005 (FDA); 2006 (EMA) |
| Duvelisib | PI3K inhibitor | PI3Kδ, PI3Kγ | CLL, SLL | 25 mg BID | 2013 (EMA;OD) 2018 (FDA; OD) |
| Encorafenib⸹ | BRAF inhibitor | B-Raf | MEL (BRAFV600+) | 450 mg QD | 2018 (EMA, FDA) |
| Entrectinib | ROS1/NTRK inhibitor | TrkA/B/C, ROS1, ALK | NSCLC (ROS1+) Solid tumours with NTRK gene fusions | 600 mg QD | 2019 (FDA; OD), Pending (EMA) |
| Erdafitinib | FGFR inhibitor | FGFR1-4, RET, CSF1R, PDGFRα/β, FLT4, KIT, VEGFR2 | UC | 8 mg QD initially, increase to 9 mg QD^˥^ | 2019 (FDA) |
| Erlotinib | EGFR inhibitor | EGFR | NSCLC (EGFR mut.) PC | 100 mg QD 100 mg QD + gemcitabine | 2004 (FDA), 2005 (EMA) |
| Everolimus | mTOR inhibitor | mTOR | BC, NET, RCC | 10 mg QD | 2009 (EMA, FDA) |
| Fedratinib | JAK/FLT3 inhibitor | JAK2, FLT3 | MF | 400 mg QD | 2019 (FDA; OD) |
| Gefitinib | EGFR inhibitor | EGFR | NSCLC (EGFR mutated) | 250 mg QD | 2003 (FDA), 2009 (EMA) |
| Gilteritinib | FLT3 inhibitor | FLT3, AXL, ALK, LTK | AML (FLT3+) | 120 mg QD | 2018 (EMA, FDA) |
| Glasdegib | HSP inhibitor | SMO | AML | 100 mg QD | 2018 (FDA; OD) Pending (EMA; OD) |
| Ibrutinib | BTK inhibitor | BTK | CLL, WM MCL | 420 mg QD 560 mg QD | 2013 (FDA; OD), 2014 (EMA; OD) |
| Idelalisib | PI3K inhibitor | PI3Kδ | CLL, FNHL | 150 mg BID | 2014 (EMA, FDA) |
| Imatinib | BCR-ABL inhibitor | BCR-ABL kinase, KIT, PDGFRα/β | ALL (Ph+)  ASM, DFSP, GIST, MDS/MPD  CEL  CML (Ph+) | 600 mg QD,  Children: 340 mg/m^2^/day  400 mg QD  100 mg QD CP: 400 mg QD AP, BC: 600 mg QD Children: 340 mg/m^2^/day | 2001 (EMA, FDA) |
| Ixazomib | Proteasome inhibitor | 20S-Proteasome | MM | 4 mg QW (on days 1, 8 and 15 of 28-day cycle) | 2015 (FDA; OD) 2016 (EMA; OD) |
| Lapatinib | EGFR inhibitor | EGFR, HER2 | BC (ER^+/-^, HER2^+^) | 1250 mg QD (with capecitabine) 1000 mg QD (with trastuzumab) 1500 mg QD (with AI) | 2007 (FDA), 2008 (EMA) |
| Larotrectinib | NTRK inhibitor | TrkA/B/C | Solid tumours with NTRK gene fusions | 100 mg BID (Children: 100 mg/m^2^ BID; max 100 mg BID) | 2018 (FDA; OD) 2019 (EMA) |
| Lenvatinib | VEGF inhibitor | VEGFR1-3, FGFR1-4, RET, KIT, PDGFRα | HCC  DTC  RCC | 8 mg QD (<60 kg),  12 mg QD (≥60 kg) 24 mg QD  18 mg QD (+ everolimus 5 mg QD) | 2015 (EMA, FDA) |
| Lorlatinib | ALK inhibitor | ALK, ROS1, TYK1, FER, FPS, TrkA/B/C, FAK, FAK2, ACK | NSCLC (ALK rearr.) | 100 mg QD | 2018 (FDA), 2019 (EMA) |
| Midostaurin | FLT3 inhibitor | FLT3, KIT | AML (FLT3+)  ASM | 50 mg BID  100 mg BID | 2017 (EMA, FDA; OD) |
| Neratinib | EGFR inhibitor | EGFR, HER2, HER4 | BC (ER^+/-^, HER2^+^) | 240 mg QD | 2017 (FDA), 2018 (EMA) |
| Nilotinib | BCR-ABL inhibitor | BCR-ABL kinase, KIT, PDGFRα/β | CML (Ph+) | Newly diagnosed: 300 mg BID CP, AP or BC with resistance to prior therapy: 400 mg BID Children: 230 mg/m^2^ | 2007 (EMA, FDA; OD) |
| Nintedanib | VEGF inhibitor | VEGFR1-3, PDGFRα/β, FGFR1-3, FLT3, Lck, Src | NSCLC | 200 mg BID on days 2-21 of docetaxel q3w cycle | 2014 (EMA) |
| Osimertinib | EGFR inhibitor | EGFR T790M | NSCLC (EGFR mut.) | 80 mg QD | 2015 (FDA), 2016 (EMA) |
| Palbociclib | CDK inhibitor | CDK4/6 | BC (ER^+^, HER2^-^) | 125 mg QD for 3 weeks on, 1 week off | 2015 (EMA, FDA) |
| Panobinostat | HDAC inhibitor | HDAC | MM | 20 mg QD on days 1,3,5,8,10 and 12 of 21-day cycle | 2015 (EMA, FDA; OD) |
| Pazopanib | VEGF inhibitor | VEGFR1-3, FGFR2, KIT, PDGFRα/β | RCC, STS | 800 mg QD | 2009 (FDA), 2010 (EMA) |
| Pexidartinib | CSF1R inhibitor | CSF1R, KIT, FLT3 | TGCT | 400 mg BID | 2019 (FDA; OD) Pending (EMA; OD) |
| Ponatinib | BCR-ABL inhibitor | BCR-ABL kinase, FGFR1-3, FLT3, VEGFR2, KIT, RET, PDGFRα | ALL (Ph+), CML (Ph+) | 45 mg QD | 2012 (FDA; OD) 2013 (EMA; OD) |
| Regorafenib | VEGF inhibitor | VEGFR1-3, KIT, PDGFRβ, RAF, RET, | CRC, GIST, HCC | 160 mg QD for 3 weeks on, 1 week off | 2012 (FDA), 2013 (EMA) |
| Ribociclib | CDK inhibitor | CDK4/6 | BC (ER^+^, HER2^-^) | 600 mg QD for 3 weeks on, 1 week off | 2015 (EMA, FDA) |
| Ruxolitinib | JAK inhibitor | JAK1/2 | MF PV | 15 mg BID 10 mg BID | 2011 (FDA), 2012 (EMA) |
| Sorafenib | VEGF inhibitor | VEGFR2/3, FLT3, FGFR1, KIT, PDGFRβ, RAF, RET | DTC, HCC, RCC | 400 mg BID | 2005 (FDA; OD), 2006 (EMA; OD) |
| Sunitinib | VEGF inhibitor | VEGFR1-3, KIT, PDGFRα/β, FLT3, RET | RCC  GIST, NET | 50 mg QD for 4 weeks on,  2 weeks off  37.5 mg QD, continuously | 2006 (EMA, FDA) |
| Tivozanib | VEGF inhibitor | VEGFR1-3, KIT | RCC | 1340 µg QD for 3 weeks on, 1 week off | 2017 (EMA) |
| Trametinib† | MEK inhibitor | MEK1-2 | ATC, MEL, NSCLC  (if BRAFV600+) | 2 mg QD | 2013 (FDA), 2014 (EMA) |
| Vandetanib | VEGF inhibitor | VEGFR2, EGFR, RET | MTC | 300 mg QD | 2011 (FDA), 2012 (EMA) |
| Vemurafenib⸸ | BRAF inhibitor | B-Raf | MEL (BRAFV600+) | 960 mg BID | 2011 (FDA), 2012 (EMA) |
| Vismodegib | HSP inhibitor | SMO | BCC | 150 mg QD | 2012 (FDA), 2013 (EMA) |
| Vorinostat | HDAC inhibitor | HDAC | CTL | 400 mg QD | 2006 (FDA; OD) |

⸹ When used in combination therapy, encorafenib is used together with binimetinib.
† When used in combination therapy, dabrafenib is used together with trametinib.
⸸ When used in combination therapy, vemurafenib is used together with cobimetinib.
˥ Only if serum phosphate < 5.5 mg/dL on days 14-21. **Abbreviations:
General:** AP: accelerated phase; BP: blast phase; CP: chronic phase; BID: twice daily; EMA: European medicines agency; FDA: U.S. Food & Drug Administration; mut.: mutated; OD: orphan drug; QD: once daily; rear.: rearranged.
**Compound classes/molecular targets:** ACK: activated CDC42 kinase; ALK: anaplastic lymphoma kinase; Bcl-2: B-cell lymphoma 2; BCR-ABL: Breakpoint cluster region-Abelson; BTK: Bruton’s tyrosine kinase; CDK4/6: cycline-dependent kinases 4 and 6; CSF1R: colony stimulating factor 1 receptor; EGFR: epidermal growth factor receptor; EphA2: Ephrin type-A receptor 2; ER: estrogen receptor; FAK: focal adhesion kinase; FGFR: fibroblast growth factor receptor; FLT3: FMS-related tyrosine kinase 3; HDAC: histone deacetylase; HER: human epidermal growth factor receptor; HSP: hedgehog-signalling pathway; IGF-1R: insulin-like growth factor 1 receptor; JAK: Janus kinase; KIT: mast/stem cell growth factor receptor; Lck: lymphocyte-specific protein tyrosine kinase; LTK: Leukocyte receptor tyrosine kinase; MEK: mitogen-activated protein kinase; mTOR: mammalian target of rapamycin; NTRK: neurotrophic tyrosine receptor kinase; PDGFR: platelet-derived growth factor receptor; Ph+: philadelphia-chromosome positive; PI3K: phosphoinositide 3-kinase; RAF: RAF kinase family comprising A-Raf, B-Raf and c-Raf; RET: “rearranged during transfection” proto-oncogene; ROS1: c-ros oncogene 1; SMO: smoothened transmembrane protein; Src: gene product of SRC, Proto-oncogene tyrosine-protein kinase Src; Trk: tropomyosin receptor kinase; TYK: tyrosine kinase; VEGF(R): vascular endothelial growth factor (receptor).
**Indications:** ALL: acute lymphoblastic leukaemia; AML: acute myeloid leukaemia; ASM: aggressive systemic mastocytosis; ATC: anaplastic thyroid cancer; BC: breast cancer; BCC: basal cell carcinoma; B-Raf: gene product of BRAF, serine/threonine-protein kinase B-Raf; CEL: chronic eosinophilic leukaemia; CLL: chronic lymphocytic leukaemia; CML: chronic myeloid leukaemia; CRC: colorectal cancer; CTL: cutaneous T-cell lymphoma; DFSP: dermatofibrosarcoma protuberans; DTC: differentiated thyroid cancer; FNHL: follicular B-cell non-Hodgkin lymphoma; GIST: gastrointestinal stromal tumours; HCC: hepatocellular carcinoma; MCL: mantle cell lymphoma; MDS/MPD: myelodysplastic/myeloproliferative diseases; MEL: melanoma; MF: myelofibrosis; MM: multiple myeloma; MTC: medullary thyroid cancer; NET: neuroendocrine tumours; NSCLC: non-small cell lung cancer; PC: pancreatic cancer; PV: polycythaemia vera; RCC: renal cell carincoma; SLL: small lymphocytic lymphoma; STS: soft-tissue sarcoma; TC: thyroid cancer; TGCT: tenosynovial giant cell tumour; UC: urothelial carcinoma; WM: Waldenstroem’s macroglobulinaemia

**Supplementary Table 1b: Overview of oral targeted antihormonal drugs (AHDs)**

| Compound name | Compound class | Molecular targets | Indication | Approved dose | First approval [year] |
| --- | --- | --- | --- | --- | --- |
| Abiraterone | CYP17 inhibitor | CYP17 | PCa | 1000 mg QD | 2011 (EMA, FDA) |
| Anastrozole | AI | Aromatase | BC (ER^+^, HER2^-^) | 1 mg QD | 1995 (FDA), 1996 (GER) |
| Apalutamide | ARA | AR | PCa | 240 mg QD | 2018 (FDA), 2019 (EMA) |
| Darolutamide | ARA | AR | PCa | 600 mg BID | 2019 (FDA), Pending (EMA) |
| Bicalutamide | ARA | AR | PCa | 50 mg QD (metastatic) 150 mg QD (locally advanced) | 1995 (FDA), 1996 (GER) |
| Enzalutamide | ARA | AR | PCa | 160 mg QD | 2012 (FDA), 2013 (EMA) |
| Exemestane | AI | Aromatase | BC (ER^+^, HER2^-^) | 25 mg QD | 1999 (GER, FDA) |
| Letrozole | AI | Aromatase | BC (ER^+^, HER2^-^) | 2.5 mg QD | 1997 (GER, FDA) |
| Tamoxifen | SERM | ER | BC (ER^+^, HER2^-^) | 20 mg QD | 1977 (FDA), 1984 (GER) |

**Abbreviations:** AI: aromatase inhibitor; AR: androgen receptor; ARA: androgen receptor antagonist; BC: breast cancer; BID: twice daily; CYP: cytochrome-P450; EMA: European medicines agency; ER: estrogen receptor; FDA: U.S. Food & Drug Administration; GER: Germany; HER: human epidermal growth factor receptor; PCa: prostate cancer; QD: once daily; SERM: selective estrogen receptor modulator.

**Supplementary Table 1c: Overview of other targeted oral antineoplastic drugs (OADs)**

| Compound name | Compound class | Molecular targets | Indication | Approved dose | First approval [year] |
| --- | --- | --- | --- | --- | --- |
| Enasidenib | IDH inhibitor | IDH2 | AML | 100 mg QD | 2017 (FDA; OD) Pending (EMA; OD) |
| Ivosidenib | IDH inhibitor | IDH1 | AML | 500 mg QD | 2018 (FDA; OD) Pending (EMA; OD) |
| Niraparib | PARP inhibitor | PARP1-2 | OC | 300 mg QD | 2017 (EMA, FDA; OD) |
| Olaparib | PARP inhibitor | PARP1-3 | BC, OC | 300 mg BID (tablets) **or**  400 mg BID (capsules) | 2014 (EMA; FDA) |
| Rucaparib | PARP inhibitor | PARP1-2 | OC | 600 mg BID | 2016 (FDA), 2018 (EMA) |
| Talazoparib | PARP inhibitor | PARP1-2 | BC | 1 mg QD | 2018 (FDA), 2019 (EMA) |
| Venetoclax | Bcl-2 inhibitor | Bcl-2 | AML, CLL | 20 mg QD initial; increase stepwise to 400 mg QD | 2016 (EMA, FDA) |

**Abbreviations:** AML: acute myeloid leukaemia; Bcl-2: B-cell lymphoma 2; BC: breast cancer; BID: twice daily; CLL: chronic lymphocytic leukaemia; EMA: European medicines agency; FDA: U.S. Food & Drug Administration; IDH: isocitrate dehydrogenase; OD: orphan drug; OC: ovarian cancer; PARP: poly(ADP-ribose)polymerase; QD: once daily. Sources of information: Summaries of Product Characteristics (SmPCs) (EMA) [97] and drug labels of marketed compounds (FDA) [2].

**Supplementary Figure 1: Overview of oral antineoplastic drugs and their targets**

**
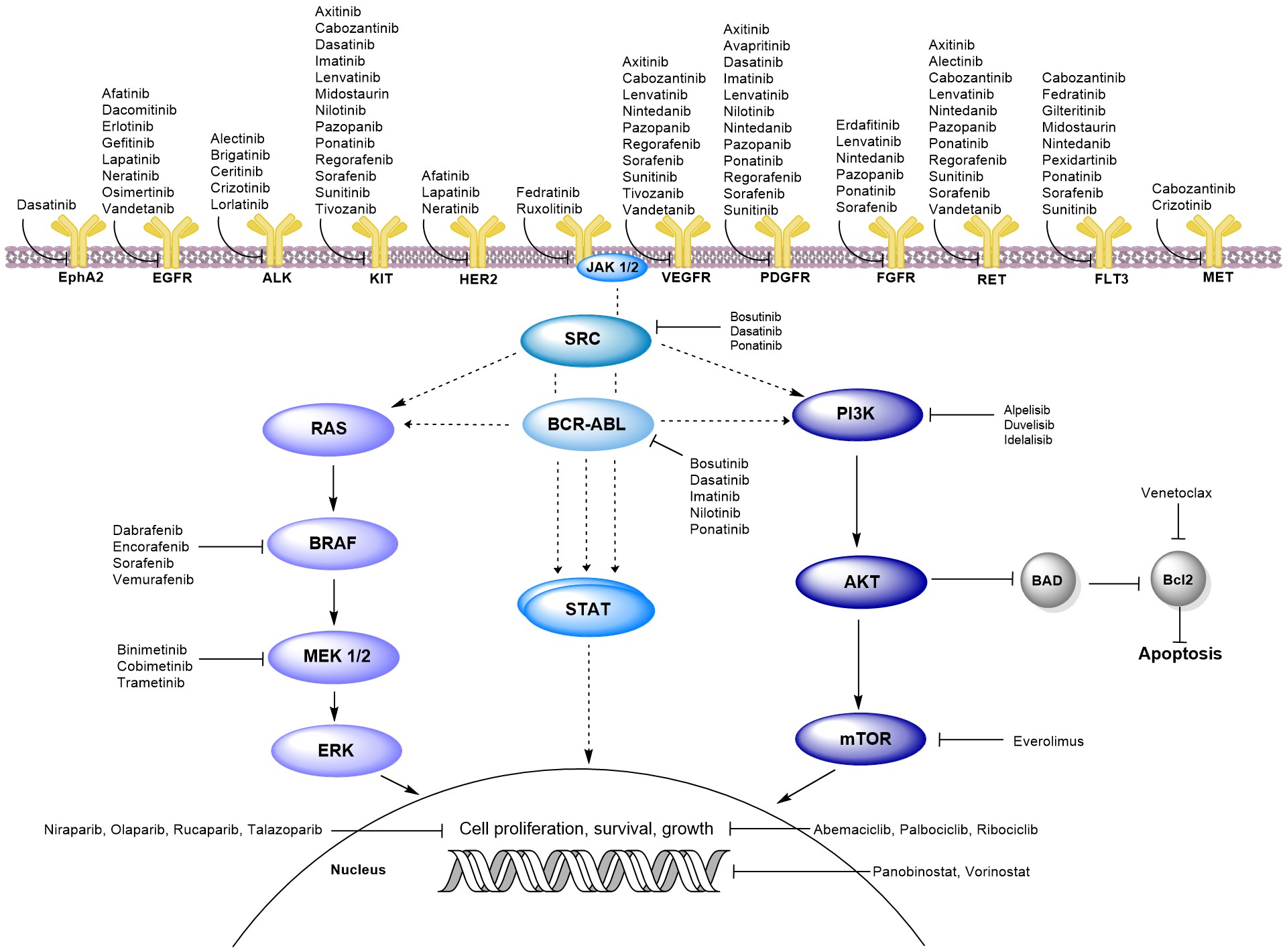
**

**
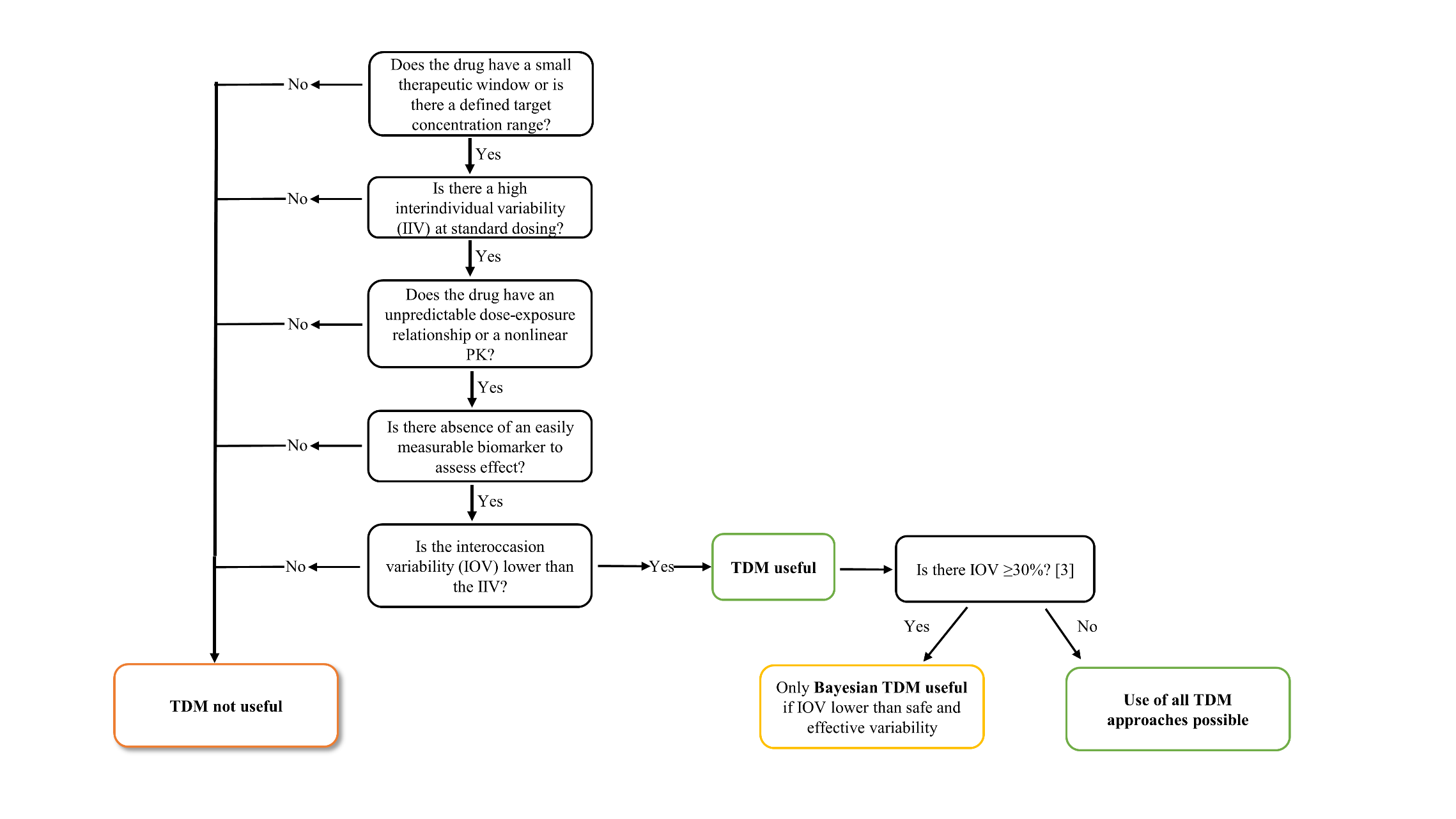
Supplementary Figure 2: TDM flow scheme** [3]

**References**

[1] Head of Medicines Agency HumanMRIndex. http://mri.medagencies.org/Human/. Accessed 28 March 2019

[2] Food and Drug Administration Online Label Repository. https://labels.fda.gov/. Accessed 28 March 2019

[3] Abrantes JA, Jönsson S, Karlsson MO, Nielsen EI (2019) Handling interoccasion variability in model-based dose individualization using therapeutic drug monitoring data. Br J Clin Pharmacol 85:1326–1336. https://doi.org/10.1111/bcp.13901
